# Evaluating the Role of Susceptibility Inducing Cofactors and of Acetaminophen in the Etiology of Autism Spectrum Disorder

**DOI:** 10.3390/life14080918

**Published:** 2024-07-23

**Authors:** John P. Jones, Lauren Williamson, Zacharoula Konsoula, Rachel Anderson, Kathryn J. Reissner, William Parker

**Affiliations:** 1 WPLab, Inc., Durham, NC 27707, USA; jpjones@williamparkerlab.org (J.P.J.III); zkonsoula@williamparkerlab.org (Z.K.); rachel.anderson@williamparkerlab.org (R.A.); 2 Department of Biological Sciences, Northern Kentucky University, Highland Heights, KY 41099, USA; williamsol6@nku.edu; 3 Department of Psychology and Neuroscience, University of North Carolina, Chapel Hill, NC 27599, USA; kreissne@email.unc.edu

**Keywords:** acetaminophen, oxidative stress, paracetamol, autism spectrum disorder

## Abstract

More than 20 previously reported lines of independent evidence from clinical observations, studies in laboratory animal models, pharmacokinetic considerations, and numerous temporal and spatial associations indicate that numerous genetic and environmental factors leading to inflammation and oxidative stress confer vulnerability to the aberrant metabolism of acetaminophen during early development, leading to autism spectrum disorder (ASD). Contrary to this conclusion, multivariate analyses of cohort data adjusting for inflammation-associated factors have tended to show little to no risk of acetaminophen use for neurodevelopment. To resolve this discrepancy, here we use in silico methods to create an ideal (virtual) population of 120,000 individuals in which 50% of all cases of virtual ASD are induced by oxidative stress-associated cofactors and acetaminophen use. We demonstrate that Cox regression analysis of this ideal dataset shows little to no risk of acetaminophen use if the cofactors that create aberrant metabolism of acetaminophen are adjusted for in the analysis. Further, under-reporting of acetaminophen use is shown to be a considerable problem for this analysis, leading to large and erroneously low calculated risks of acetaminophen use. In addition, we argue that factors that impart susceptibility to acetaminophen-induced injury, and propensity for acetaminophen use itself, can be shared between the prepartum, peripartum, and postpartum periods, creating additional difficulty in the analysis of existing datasets to determine risks of acetaminophen exposure for neurodevelopment during a specific time frame. It is concluded that risks of acetaminophen use for neurodevelopment obtained from multivariate analysis of cohort data depend on underlying assumptions in the analyses, and that other evidence, both abundant and robust, demonstrate the critical role of acetaminophen in the etiology of ASD.

## 1. Introduction

Evidence regarding a causal relationship between early life exposure to acetaminophen and autism spectrum disorder (ASD) has been reviewed recently by our group [1,2,3]. Twenty-two distinct lines of evidence have been described (see Table 1 in Parker et al. [3]), allowing us to conclude with no reasonable doubt that factors leading to susceptibility combined with acetaminophen exposure cause many if not most cases of ASD. That evidence will be summarized in this Introduction. While no one association or other line of evidence is sufficient to reach any firm conclusion, we view the weight of evidence, when taken together, as overwhelming [1,2,3].

The first study reporting an association between acetaminophen and ASD, published in 2008 by Stephen Schultz, used a case-controlled methodology and showed that acetaminophen (odds ratio (OR) = 8.23; 95% confidence interval (CI) = 1.56–43.3; *p* = 0.013) but not ibuprofen use (OR = 0.89; CI = 0.10–8.30; *p* = 0.918) following an adverse reaction to a vaccination is associated with regressive ASD [4]. The Schultz study also showed that acetaminophen use itself, regardless of vaccination, was strongly associated with regressive ASD (OR = 20.9; CI = 1.33–32.9; *p* = 0.031) [4]. Some have criticized the 2008 study as potentially biased in terms of participant selection, which recruited individuals using advertising on the internet. However, a retrospective assessment of information available at the time the survey was conducted [2] shows that the parents in the study were likely biased against vaccines as a cause of their children’s ASD, providing a very useful sample for evaluation of the role of acetaminophen in their children’s ASD. The results of the Schultz study thus provide an explanation for the common belief among parents that vaccines cause ASD [5,6].

In addition, associations between acetaminophen use and ASD have been found in South Korea (discussed by our group in Patel et al. [1]), Israel (discussed by our group in Patel et al. [1]), and Scandinavian countries (discussed by our group in Parker et al. [3]). The strong association of ASD with cord blood acetaminophen metabolites (OR = 3.62; CI = 1.62–8.60 for the third compared to the first tertile) [7], and with circumcision (OR infantile ASD = 2.06; CI = 1.36–3.13) [8], a minor medical procedure often performed with acetaminophen use, provides further compelling evidence. Additional evidence is provided by several studies in adult humans showing that acetaminophen specifically impairs social awareness [9,10,11], which is impaired in individuals with ASD.

The belief that acetaminophen is safe in babies and/or children when used as directed is reported in thousands of peer-reviewed publications, but this belief was found to be based on the false assumption that babies process drugs, and specifically acetaminophen, in the same manner as adults [12]. The effects of acetaminophen on neurodevelopment were never evaluated prior to its approval for use during early neurodevelopment, despite the fact that its action alters brain metabolism [13] and signaling [14,15]. Studies in laboratory mice evaluating the effects of acetaminophen exposure during early postnatal life were first described in 2014, and they demonstrated profound impairment of learning ability during adulthood (2 months of age) following acetaminophen exposure (2 doses of 30 mg/kg) on postnatal day 10 [16].

Studies from five independent laboratories using laboratory rats and mice have now been published [16,17,18,19,20,21,22], demonstrating that acetaminophen, even when administered during pregnancy or to young animals at near [16] or even below [20,21] currently accepted clinical doses (14.7 mg/kg single dose, increasing with repeated use depending on pharmacokinetics), alters long term behaviors involved in memory and social function. In addition, work by McCarthy and colleagues [23] has shown that acetaminophen and other small molecules with similar effects on lipid metabolism cause changes that are associated with cerebellar atrophy in males more so than in females. Thus, the drug could never have passed pre-clinical testing and been approved for even experimental use in babies and small children under current FDA guidelines (discussed by our group in Patel et al. [1]). Further, a study in adult laboratory animals shows that higher doses of acetaminophen (250 mg/kg) result in the death of cortical neurons [24].

### 1.1. An Actual Rise in the Prevalence of ASD

The rise in prevalence of ASD is associated temporally with the rise in the use of acetaminophen. The introduction of the analgesics phenacetin and acetanilide in the late 1880s, which are both metabolized by the body into acetaminophen, was followed by the discovery of ASD in the 1920s [25] and rediscovery in the 1940s [26,27,28]. Following the approval of acetaminophen for pediatric use in the 1950s, the incidence of ASD began to rise slowly. The incidence of ASD began to rise more rapidly beginning in the 1980s, after acetaminophen replaced aspirin due to concerns over Reye’s syndrome, and accelerated during the 1990s and 2000s at a time when between 115 and 250 million dollars (200–475 million dollars/year with inflation adjustment for 2024) in direct-to-consumer advertising per year encouraged acetaminophen use [29,30].

The rise in the prevalence of ASD is clearly affected by increasing awareness, social programs, funding, changing diagnostic criteria, and potentially other factors. However, investigators in 1987 were keenly aware of these issues at that time, when the documented prevalence of ASD had reached one in 645 children. As stated by Matsuishi et al. [31]:

“What is the reason for the high (1 in 645) prevalence rates? We doubt that differences in diagnostic criteria and survey methods account for these high rates, although such factors may explain the large differences between the studies done between 1966 and 1980 and the studies done since 1983. In fact, the earlier studies were carefully performed using good survey methods. Although in some studies the diagnostic criteria may have been different, the diagnostic criteria were similar in the study by Hoshino et al. (Fukushima J Med Sci 1979;26:31-42.) and in our study, and both studies were done in Japan in similar cultural, ethnic, and socioeconomic regions. However, our prevalence is three times that of Hoshino et al. Could the real prevalence of autism be increasing in the world?”

Matsuishi and colleagues were at a loss as to the cause of the increase, speculating that “the survival of brain-impaired babies” may contribute to the increase [31]. Almost 20 years later, in 2002, with the highest recorded prevalence of ASD at 1/175, Williams and colleagues noted that the rising prevalence at that time was not clearly explained by changes in diagnostic criteria:

“…it was not possible to account entirely for the effect of the diagnostic criteria on the prevalence estimates as the ICD-10 and DSM-IV diagnostic schema leave some scope for variation in their interpretation and application.”

Investigators have, historically, been keenly aware of social and medical factors that might affect measures of ASD prevalence, and many yet believed that some environmental factors must be at play in the actual etiology of ASD. Indeed, as Coury and Nash noted in 2003 [32]:

“If MMR and thimerosal [factors related to vaccination] aren’t responsible, what’s the causative factor for autism? The simple answer is that it is not known.”

In the 20 years since Coury and Nash wrote their article, the prevalence of ASD has continued to climb. It could be argued that, despite the fact that investigators have been aware of the potential problem for almost four decades, early investigators were unable to conduct a reliable survey, and/or current methods and/or awareness and/or diagnostic criteria and/or other factors keep changing, resulting in an increased prevalence of ASD with much less or even no increase in actual incidence. However, available evidence pertinent to this issue includes a rich and diverse array of information, all of which should be included when considering the changing prevalence of ASD though time. For example, the circumcision-associated prevalence of infantile ASD [8], noted above, suggests that some factor or factors can indeed cause a true increase in the incidence of ASD, especially in newborns who are most susceptible to drug-induced toxicity [33]. Although it is impossible to go back in time and re-assess the prevalence of ASD in the past using modern techniques, ASD has been described as relatively rare compared to other disorders affecting mental health in areas of the world experiencing shortages of even the most basic medicines. For example, Victor Lotter, working in six African countries from 1975 to 1976, expected 5% to 8% of “severely subnormal” children to have “marked autistic behaviour” based on studies in Britain, but identified ASD behaviors in only 5 of more than 400 (<1.25%) such children [34]. In another example, Hyun Uk Kim evaluated attitudes about ASD in Leon, Nicaragua, in 2010, a country plagued by shortages of even the most basic medications for decades [35,36]. She found almost no awareness of ASD even among educators, and a ratio of Down syndrome to ASD of 33 to 1 in a school for special needs children. Based on current prevalence rates in the US, the ratio of Down syndrome to ASD is expected to be about 1 to 19 (1 in 691 or 0.14% with Down syndrome [37] and 1 in 36 or 2.8% with ASD [38]) rather than 33 to 1. In addition, Somali immigrants living in North America view ASD as a Western disease, with many believing that it does not exist in their native country [39]. One explanation for these observations is that the absence of medication, particularly acetaminophen, results in a lower incidence of ASD.

Based on overwhelming evidence, one environmental factor that induces ASD, albeit only in susceptible children, has been identified as acetaminophen. The drug fits the description of the factor sought by Matsuishi in 1987, and by Coury and Nash in 2003. Further, since drugs that are metabolized into acetaminophen by the human body have been commercially available for over a century (discussed by our group in Parker et al. [3]), it is plausible that acetaminophen was the root cause of at least some cases of ASD when, in the 1940s, Kanner tragically and wrongly attributed ASD to aloof parenting [40].

### 1.2. Fetal Alcohol Spectrum Disorder as Another Example of an Analgesic-Mediated Spectrum of Developmental Injury

One of the major unknowns facing researchers in the field of ASD has been the reason for the broad spectrum of phenotypes that exist. While it is unknown why acetaminophen-mediated neurodevelopmental injury is expressed in such a broad fashion, it is known that developmental injury mediated by another drug, alcohol, causes a spectrum of disorders classified under the umbrella of fetal alcohol spectrum disorder (FASD). FASD shares numerous similarities with ASD. Because both disorders are characterized by a spectrum of conditions, and because neither disorder can be diagnosed by an objective test or biomarker, some difficulty with diagnosis of both disorders is evident [41,42]. Both disorders are highly heterogeneous in terms of cognitive deficits, such as sensory and motor difficulties [43,44], attention deficient hyperactively disorder-like symptoms, including executive dysfunction, attentional deficits, and impulsivity [45,46], along with other comorbid mental illnesses such as anxiety, mood disorders, obsessive compulsive disorders, etc. [47,48], and intellectual disability [49,50]. Both disorders are complex and are at times associated with comorbid medical conditions. For example, FASD is associated with high rates of seizures [51], sleep problems [52], abnormal eating behaviors [53], and disorders related to immune function [54], all conditions associated with ASD [55]. Further, both disorders are known to have many risk factors that contribute to susceptibility, including genetic factors [56,57] and environmental factors, such as those relating to parental age, health, nutrition, and other prenatal and postnatal factors [58,59]. The commonly recommended treatment for both disorders is early intervention [60,61], akin to rehabilitation after any other type of brain injury, although the success of the treatment is highly variable in both cases.

ASD and FASD share another important property in common. Both are caused by the exposure of susceptible individuals to drugs that have analgesic properties [62,63] and that are metabolized by the human body into toxic electrophilic compounds. Acetaminophen is metabolized into a quinoneimine [64], and alcohol is metabolized into an aldehyde [65]. The similarities between ASD and FASD provide one line of evidence that ASD is chemically induced. Although this line of evidence does not explain how a single drug can cause a spectrum of disorders, it does show that a single drug is capable of causing a spectrum of disorders. As with the other lines of evidence leading to the conclusion that ASD is induced by acetaminophen exposure in susceptible individuals, this line of evidence alone is not conclusive. Rather, this evidence fits into a larger picture that has, with time, become clear.

### 1.3. The Metabolism of Acetaminophen and the Etiology of ASD

The well-established understanding of metabolic pathways by which acetaminophen is metabolized provides some of the strongest evidence indicating that exposure of susceptible children to acetaminophen causes ASD. Indeed, the pharmacological/pharmacokinetic properties of acetaminophen are well known. Acetaminophen is metabolized via three major pathways that have been well established for decades [66]. Two pathways, glucuronidation and sulfation, result in elimination of the drug from the body without harm. The fact that glucuronidation is not adequately developed during the first 10 days of postpartum life is also well established, and was pointed out by the renowned pediatrician/scientist William L. Nyhan in his 1961 treatise arguing for the evaluation of drugs in neonatal animal models before use in neonates [33]. Further, independent observations have demonstrated that the other safe metabolic pathway for disposal of acetaminophen, sulfation, is impaired in many children with ASD [67,68,69]. The third pathway, cytochrome P450-mediated oxidation, leads to the production of a toxic metabolite, N-Acetyl-p-benzoquinone imine (NAPQI). NAPQI is a highly reactive electrophile, most of which is rapidly neutralized by glutathione within seconds under normal conditions. However, excess production of NAPQI and/or depletion of glutathione due to chronic inflammation and/or oxidative stress result in NAPQI-mediated cytotoxicity and eventual programmed cell death. Unfortunately, children with ASD have decreased glutathione concentrations [70], and observed elevations in systemic oxidative inflammation and stress markers are a hallmark feature of ASD [70,71,72,73,74]. Furthermore, autoantibodies that interfere with the cerebral folate receptor have been identified in most children with ASD [75]. Since folate is required for glutathione synthesis [76], folate deficiency and thus impairment of NAPQI detoxification may be problematic in the brains of many children with ASD. Further, the strong overlap between risk factors for ASD and factors that cause inflammation and/or oxidative stress, thus impairing the safe metabolism of acetaminophen, was reviewed in 2017 by us [77]. This overlap between risk factors for ASD and for impairment of safe acetaminophen metabolism provides one more line of evidence leading to the conclusion that exposure to acetaminophen coupled with numerous factors leading to susceptibility cause many if not most cases of ASD.

### 1.4. The Weight of Evidence and the Role of Acetaminophen in the Etiology of ASD

No single line of evidence is sufficiently compelling to lead to the conclusion that exposure of susceptible babies and children to acetaminophen induces ASD. Rather, the role of acetaminophen in the pathogenesis of ASD is illuminated by numerous lines of evidence, diverse in nature, taken together as a whole. Twenty-two lines of evidence, most listed above, were tallied in our most recent review of that evidence [3]. The apparently low prevalence of ASD in countries with chronic shortages of even the most basic medicines, which likely includes acetaminophen, is described above, and adds a 23rd line of evidence. Numerous similarities, described above, between ASD and fetal alcohol syndrome, a known effect of pathologic drug metabolism, adds a 24th line of evidence. Among the evidence lies over a dozen associations that remain unexplained if acetaminophen is not considered in the etiology of ASD [3]. Examples of observations that lack any reasonable explanation other than the involvement of acetaminophen in the pathogenesis of ASD include the association of cord blood acetaminophen with ASD (OR = 3.62; CI = 1.62–8.60 for the third compared to the first tertile) [7], the association of circumcision with more than double the incidence of infantile ASD (HR = 2.06; CI = 1.36–3.13) [8], and a case-controlled study showing that regression into ASD is associated with acetaminophen use between 12 and 18 months of age (OR = 20.9; CI = 1.33–32.9) [4]. Further, if acetaminophen is not neurotoxic during neurodevelopment, humans must be both unexpectedly and inexplicably insensitive to acetaminophen exposure during neurodevelopment compared to laboratory animals (discussed by our group in Patel et al. [1]).

Based on available evidence, the connection between ASD and acetaminophen offers an extremely clear example of an application of Occam’s Razor. This heuristic tool is considered an important element in scientific processes and advancement [78] and dictates that the simpler explanation that accounts for available observations is likely correct. A tally published in 2022 [1] found six unknown factors that must be invoked and eight largely independent observations that must be attributed to coincidence if acetaminophen is not involved in the induction of ASD. Indeed, the simplest explanation for all observations is that acetaminophen accounts for the vast majority of all cases of ASD [3].

**Figure 1:**
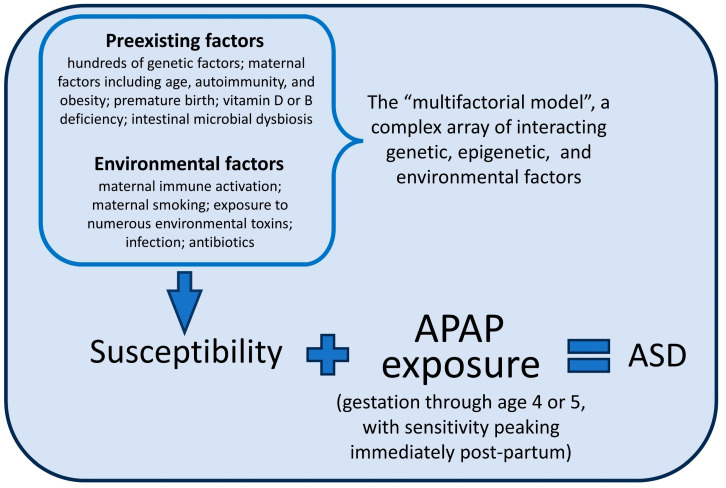
The role of acetaminophen (APAP) and of a complex array of interacting genetic, epigenetic, and environmental factors in the etiology of autism spectrum disorder (ASD). The list of preexisting and environmental factors comprising the multifactorial model is intended to be representative and is not an exhaustive list. A similar model for fetal alcohol spectrum disorder (FASD) is currently accepted, with alcohol replacing acetaminophen in that model.

A key element to any model describing the role of acetaminophen exposure in the pathogenesis of ASD is the fact that some individuals are more susceptible than others. With almost all children now exposed to acetaminophen [2], only one out of every 36 children actually acquire ASD [38]. A diagram describing the acetaminophen-induced pathogenesis of ASD is shown in Figure 1, including the contribution of a complex array of genetic, epigenetic, and environmental factors rendering individuals sensitive to acetaminophen-mediated injury. These factors lead to oxidative stress and/or chronic inflammation, lowering the body’s capacity to safely metabolize acetaminophen, leading to NAPQI-mediated damage.

**Figure 2:**
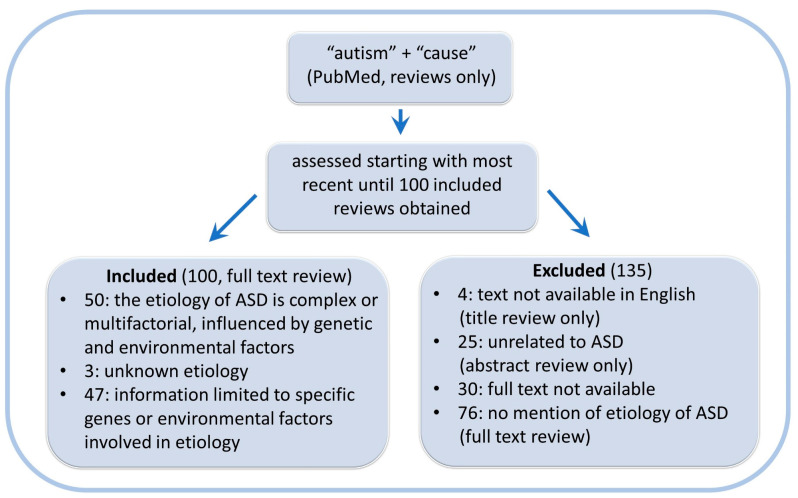
Survey of inclusion of the “multifactorial model” regarding the etiology of autism spectrum disorder (ASD) in 100 recent reviews. The survey reflects one assessment of the popularity of the multifactorial model among experts in the field, and inclusion of 100 reviews was deemed adequate for that purpose. The survey does not represent an exhaustive review of the use of a multifactorial model for ASD induction. For example, no effort was made to expand the search using terms related to “cause” (e.g., etiology, pathogenesis, induction), which may have been useful in an exhaustive review. Full texts (*n* = 30) that were not either open access or available online through the University of North Carolina, Chapel Hill Library were considered unavailable and were excluded. The survey was conducted by coauthor WP using the result of a PubMed query made 23 January 2024. The publication dates of the included reviews ranged from February 2023 to January 2024. ASD: autism spectrum disorder.

The factors rendering individuals sensitive to acetaminophen-mediated injury are highly reflective of the prevailing model of ASD pathogenesis in which a complex array of interacting genetic, epigenetic, and environmental factors contribute to the etiology of ASD. A survey of 100 literature reviews written during 2023 and 2024 revealed that half (50 out of 100) of the reviews describing an etiology or cause of ASD as multifactorial in nature (Figure 2). A small minority (3%) described the cause of ASD simply as “unknown”, and the rest (47%) limited their description of a cause to specific genetic or environmental factors, not both.

### 1.5. Multivariate Analysis of Cohort Data: Assessment of Methods by an In Silico Study

Considerable discussion has centered around the analyses of cohort data in an effort to parse out the connection between acetaminophen use during early neurodevelopment and ASD. Most recently, a study of Swedish parent/child pairs was published by Ahlqvist and colleagues, with one interpretation being that acetaminophen is safe for neurodevelopment when used as directed during pregnancy [79]. The goal of the study was to eliminate “confounding” factors in their multivariate Cox analysis to the highest extent possible, isolating and illuminating the impact of acetaminophen on neurodevelopment. The principal point highlighted in the study was that correction for effects of sibling pairs eliminated any risk attributed to acetaminophen. However, the vast majority of the raw (unadjusted for any potential confounding factor) risk was eliminated by adjustment for more than 20 inflammation-related or associated factors. That is to say, the vast majority of the risk was eliminated by adjusting for inflammation-associated factors in the analysis, not by adjustment for the sibling pairs [79].

Based on numerous independent lines of evidence, we have previously concluded that acetaminophen is relatively safe during pregnancy compared to the early postnatal period [2,3]. However, available evidence does not confirm the recently published conclusions reached by Cox analysis of the Swedish cohort data published by Ahlqvist and colleagues [79], but rather suggests that acetaminophen use during pregnancy might cause neurodevelopmental problems. Our best estimates at present place the amount of ASD induced during pregnancy at not more than 20% of all cases of ASD, and possibly 10% or less. In contrast, the conclusion reached by Ahlqvist and colleagues is that acetaminophen use during pregnancy contributes to none of the current cases of ASD [79].

Here, we use in silico (computational based/virtual) modeling to probe the impact of critical factors that undermine the conclusion reached during the recent analysis of the recent Swedish cohort data by Ahlqvist and colleagues. First, numerous “confounding” factors identified by the study team are oxidative-stress and/or inflammation-related factors that are, as described above and in Figure 1, cofactors in the induction of acetaminophen-induced injury. For example, the study team adjusted for numerous parental physical and medical conditions associated with inflammation and/or oxidative stress, including BMI, migraine headaches, other types of headaches, chronic pain, infections, fevers, any neuropsychiatric condition, and rheumatoid arthritis. They also adjusted for a wide variety of medications that are used for conditions associated with inflammatory problems, including analgesics, anti-seizure medications, and antidepressants. Further, they adjusted for social-related factors that are associated with chronic psychological stress, which are in turn associated with neuroinflammation and oxidative stress. These include smoking status, household education, income, utilization of medical resources, and cohabitation during pregnancy and at the time of delivery. As reviewed previously, many of these factors associated with inflammation and/or oxidative stress are known to be associated with ASD [77].

It is well established that cofactors or interacting variables should not be treated as independent variables when performing a Cox regression analysis [80]. That is to say, the Cox regression analysis can be used to determine whether acetaminophen or other factors cause ASD, but it cannot be used to determine if acetaminophen in combination with other factors cause ASD unless the parameters are adjusted accordingly. Specifically, the [acetaminophen + other factors] interaction parameter must be included in the analysis. At the same time, underreporting of acetaminophen use and/or an abundance of acetaminophen use by individuals not at risk can also undermine the analysis.

Here we describe an in silico model of 120,000 virtual children in which ASD is induced by acetaminophen exposure in conjunction with inflammatory/oxidative stress-related factors. The results of Cox regression analysis of the model system are presented, demonstrating the impact of adjusting for cofactors of ASD induction.

## 2. Materials and Methods

### In Silico Evaluation of the Cox Regression Model

**Figure 3:**
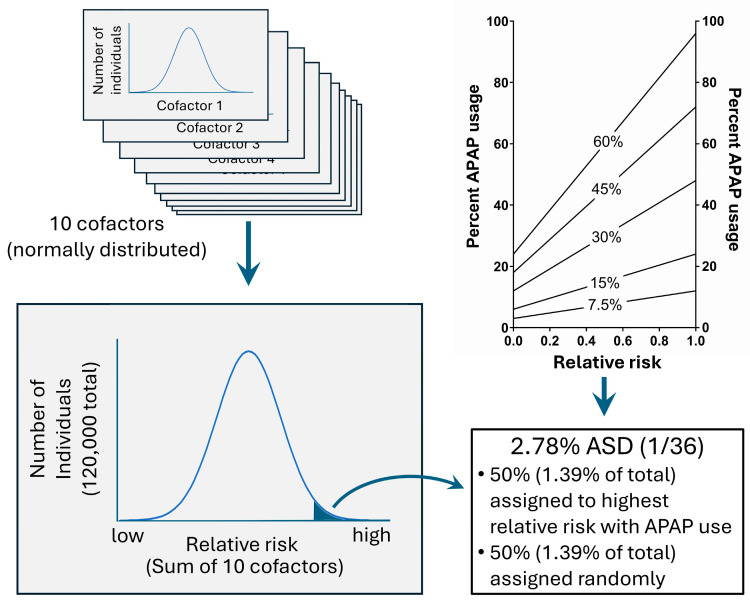
In silico modeling of the risk of ASD with acetaminophen (APAP) use. On the **top left** are 10 normally distributed risk factors that, when added together (**bottom left**), define the risk for acetaminophen-induced injury. The **top right** panel shows acetaminophen use as a function of relative risk in a virtual environment. In the **top right** panel, the *x*-axis indicates the fractional rank of risk on a linear scale, with 1 being greatest risk, 0.5 having average risk, and 0 being lowest risk. Also in the **top right** panel, the number indicated in the middle of each line indicates the average use of APAP in the virtual environment. A four-to-one ratio of APAP usage for the virtual individuals with the highest risk compared to those with the lowest risk was used (**top right** panel). The **bottom right** panel shows the final assignments of ASD in the in silico population based on risk (**bottom left** panel) and APAP exposure (**top right** panel).

For the purpose of quantitatively evaluating factors that might obfuscate the adverse neurodevelopmental effects of acetaminophen use when analyzing cohort data, an artificial (virtual, in silico) dataset was constructed with 120,000 individual (virtual, in silico) cases. Ten independent variables for each individual were randomly assigned as inflammation/oxidative stress variables. Inflammation/oxidative stress variables formed a normal distribution in the population and were generated using the software package R version 3.6.1 (R Foundation for Statistical Computing, Vienna, Austria). Acetaminophen use was assigned using a random variable function constrained such that the likelihood of exposure was defined by the sum of all 10 inflammation/oxidative stress variables, as shown in Figure 3. Ten variables defining oxidative stress and thus sensitivity to acetaminophen-induced injury may seem small given the large number of factors that might affect acetaminophen’s role in neurodevelopmental disorders. However, many independent factors, for example, a wide range of environmental toxins, may have similar effects on neuroinflammation and/or oxidative stress [77]. Thus, each of the ten factors could be considered as composites of larger numbers of factors that come together to form particular phenotypes (i.e., need for analgesic use, other neuropsychiatric conditions, socioeconomic factors, etc.) that are used in routine analyses. The ratio of acetaminophen use in virtual individuals with the highest levels of total inflammation/oxidative stress compared to those with the lowest levels of total inflammation/oxidative stress was maintained at 4 to 1. This ratio was maintained in the virtual (in silico) model to reflect the reality that individuals with the highest levels of inflammation and/or oxidative stress are more likely to use acetaminophen than individuals with the lowest levels of inflammation and oxidative stress. The model was built with no specific neurodevelopmental window in mind, and the results are equally applicable to studies obtained from data collected during any time frame, for example during pregnancy or during the first years of life.

One in 36 virtual individuals in a virtual population of 120,000 (2.78% of the total virtual population, consistent with current numbers in the US [38]) were assigned ASD, the dependent variable (Figure 3). Half of the cases of virtual ASD (affecting 1.39% of the total virtual population) were assigned to the virtual individuals with acetaminophen use and with the highest sum of all 10 inflammation/oxidative stress variables. Fifty percent of all ASD may seem conservative given that we have concluded that the vast majority of cases of ASD could be induced by acetaminophen exposure [3]. However, we have also concluded that acetaminophen exposure in the first few days postpartum is likely the time of greatest risk, with the majority of acetaminophen-induced ASD cases possibly happening at that time [3]. Further, as described in the Introduction, we previously estimated that ASD induction during pregnancy contributes to at most 20% and possibly 10% or less of all cases of ASD [3]. In addition, regressive forms of ASD likely constitute approximately a third of total ASD cases [81]. Thus, when modeling data addressing the induction of ASD during a specific time frame such as pregnancy or during early childhood, 50% is an unrealistically high value, but is nonetheless useful in evaluating the performance of a Cox analysis assessing risk factors for ASD induction. The other half of ASD cases were assigned randomly and independently of all other variables, as shown in Figure 3. Assignment of acetaminophen use and ASD were carried out using Microsoft Excel (Microsoft^®^ Excel^®^ for Microsoft 365 MSO (Version 2406 Build 16.0.17726.20078) 64-bit).

Cox regression analysis was performed using R version 4.0 (R Foundation for Statistical Computing, Vienna, Austria), along with the packages “survival” version 3.5.8 and “survminer” version 0.4.9. The time variable was set at random to effectively remove it as a factor affecting the results. Similar results were obtained when the time variable was set as a constant (see Section 3). Several factors that potentially confound real data (sex, time of diagnosis, the presence of siblings) were not relevant to the current analysis, which aimed to assess the effect of (a) adjustment for predisposing factors (cofactors) causing susceptibility to acetaminophen-induced injury and (b) the effect of underreporting of acetaminophen use. Therefore, the modeling of sex, the effects of cohorts separated by time or geography, and the presence of siblings was not conducted.

## 3. Results

### 3.1. The Effect of Treating Cofactors (Predisposing Factors) as Confounding Factors in a Cox Regression Analysis

The results of a Cox regression analysis of the in silico data with 60% use of acetaminophen and 2.78% ASD in the artificial population are shown in Table 1. In this dataset, 50% of ASD is induced by high oxidative stress combined with acetaminophen use, and 50% is randomly assigned without regard for oxidative stress or acetaminophen use (see Section 2 for details). The calculated raw (unadjusted for any variable) hazard ratio (HR), with no correction for any cofactors, was 2.461 (CI = 2.265–2.675), close to the actual hazard ratio of 2.5000 that is built into the dataset during its construction. However, as expected [80], when three out of ten of the variables defining oxidative stress were included in the model, the calculated HR was reduced to 1.844 (CI = 1.694–2007), reducing the calculated risk by almost half of the actual risk built into the construct. At the same time, HRs for the inflammatory/oxidative stress-inducing cofactors that defined susceptibility to acetaminophen were consistently about 1.2, with significant *p*-values (Table 1). The results of adjusting for nine out of ten cofactors when performing the Cox analysis were more dramatic. The calculated HRs for the cofactors remained approximately 1.2, with significant *p*-values, but the calculation showed that acetaminophen was slightly protective, with a HR of 0.905 (CI = 0.828–0.988, *p* = 0.0264).

The above results were obtained when cofactors for ASD induction (oxidative stress/inflammatory related parameters) were described as normally distributed, continuous variables. Calculations were also performed when those cofactors for ASD induction were treated as categorical variables (low, medium, and high). This change does not affect the actual HR for diagnosis with virtual ASD as a result of virtual acetaminophen use (HR set at 2.5000). When the Cox analysis was adjusted for nine out of ten categorical cofactors, the HR was 1.119, more than 12-fold lower than the actual risk. Although the risk was statistically significant, with a CI = 1.025–1.222 (*p* = 0.0123), the confidence interval was within 3% of no risk (HR = 1.0).

### 3.2. The Effects of Under-Reported Acetaminophen Use during Pregnancy in a Cox Regression Analysis

The recent analysis of acetaminophen use in the Swedish cohort during pregnancy described above [79] found a 7.5% use of acetaminophen reported in the database, which is much lower than current estimates of adult use of that drug, which tend to be in the range of 50% to 65% in North America and Europe [82,83]. Further, in a study of over 700 Swedish women during pregnancy, 59.2% of women were found to have used acetaminophen before the first 8 to 13 weeks of pregnancy [84]. In addition, a survey (8302 participants, 42% response rate) of Swedish households found that analgesics and antipyretics were the most common over-the-counter medications present in the home, with 90% of households having drugs in that category [85]. Thus, the recent analysis of the Swedish cohort may have incorporated under-use of acetaminophen into their analysis, which is expected to affect the results. To test the magnitude of this predicted effect in our model, we incorporated under-reporting of acetaminophen use into the model.

As shown in Table 2, when 60% of virtual individuals used acetaminophen, but only 7.5% were documented (virtual) users, the raw (unadjusted for any variable) calculated risk for ASD with acetaminophen use was almost 4-fold lower than the actual risk, with a calculated increased risk of only 36% (HR = 1.36) and an actual (virtual) increased risk built into the data construct of 150% (HR = 2.5000). Correction for cofactors in combination with under-documenting acetaminophen use combined to obfuscate the actual risks of acetaminophen use. When only 7.5% of virtual acetaminophen use was documented, with 60% actual virtual use, and nine out of ten cofactors were considered in the Cox analysis, no risk of acetaminophen use was found (HR = 0.992; CI = 0.885–1.111; *p* = 0.886).

Unless otherwise noted, all results were obtained with the time variable set at random to eliminate this factor from the analysis. With the time variable set at a constant, results using the parameters described above (60% virtual use of acetaminophen, 7.5% documented use, and 9 out of 10 cofactors adjusted for in the analysis) were similar (HR = 0.976; CI = 0.871–1.094; *p* = 0.677) to those obtained with the time set randomly (HR = 0.992; CI = 0.885–1.111; *p* = 0.886), suggesting that the approach used to remove the effect of time from the Cox analysis was not critical to the overall results.

**Figure 4:**
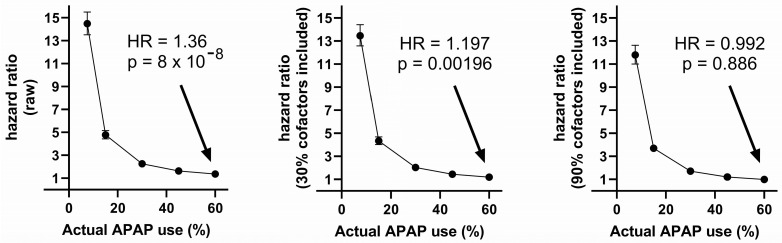
Results of Cox regression analysis with variable actual acetaminophen (APAP) use, 7.5% documented use, and 50% of all ASD induced by APAP. The fraction of actual APAP use documented in the analysis has the most profound effect on the calculated hazard ratios, although it is the inclusion of more cofactors in the Cox analysis that causes the HR to become insignificant.

The interaction between under-reporting of acetaminophen use and adjusting for cofactors in the Cox analysis is shown in Figure 4. As shown in the Figure, a Cox analysis can detect the impact of acetaminophen on the induction of virtual ASD when 50% of the virtual ASD is induced by acetaminophen. However, the real risk is only detected if the use of acetaminophen is accurately reported and cofactors are not considered in the Cox analysis. Risk, albeit lower than the actual risk built into the model, is always detected in the raw analysis (no adjustment for cofactors), even when acetaminophen use is underreported (Figure 4, left panel).

## 4. Discussion

A summary of the factors inherently limiting the multivariate analysis of cohort data to assess the role of acetaminophen in the etiology of ASD is shown in Table 3. The present study demonstrates clearly that, if the interaction of acetaminophen with a complex array of factors causes ASD, a multivariate analysis of cohort-derived data treating cofactors as confounding factors is of very limited usefulness. The present study does not, of course, demonstrate that acetaminophen plays any role in the etiology of ASD. Rather, the critical role of acetaminophen in the etiology of ASD is demonstrated by two dozen lines of evidence summarized in the Introduction.

We have previously used in silico modeling to demonstrate that increased use of acetaminophen among healthy individuals not at risk of acetaminophen-mediated ASD will lead to aberrant results in a multivariate regression analysis, eliminating the actual (virtual) impact of acetaminophen on the induction of virtual ASD from the calculated results [1]. In that previous work, the model contained 12,000 simulated children (10-fold less than the current simulation) and was adjusted for 5 out of 10 cofactors mediating sensitivity to acetaminophen-induced injury. In this analysis, we increased the number of virtual children by a factor of 10, improving our ability to detect virtual adverse drug effects. Nevertheless, we demonstrate the previously reported effect of treating cofactors as confounding factors in a Cox analysis [80], showing that adjusting for 9 out of 10 cofactors for acetaminophen-induced injury results in a loss of detection of real risks. Even when 50% of all cases of virtual ASD were induced by acetaminophen, correcting for cofactors using a Cox analysis completely eliminated the actual risk from the calculated risk. When cofactors were listed as categorical rather than continuous, some statistically significant risk did remain following adjustment for 9 out of 10 cofactors (HR = 1.119; CI = 1.025–1.222; *p* = 0.0123). However, this low level of risk, with a confidence interval closely approaching no effect (HR = 1.0), belies the fact that, in this virtual model, acetaminophen use caused 50% of all cases of virtual ASD. Such erroneously low HRs, when published as the results of analysis of actual cohort data, can lead to underappreciation of the dangers of acetaminophen use during neurodevelopment. Indeed, as described in a court’s decision to vacate a US federal lawsuit over alleged damages caused by acetaminophen use during pregnancy, the court supported the assertion that “an odds ratio between 1 and 2 is deemed low” [86]. Although the conditions under which this subjective statement could be valid are not clear, and although the statement is certainly not valid if the risk factor and the outcome in question are widespread, such views are not surprising given that so many individual risk factors associated with ASD fall within this “low” range. Indeed, all of the 10 factors in our virtual model that define risk for acetaminophen-induced ASD (50% of all ASD in this virtual model) have HRs for ASD near 1.2 in the Cox analyses.

Adjustment for interacting factors in a Cox regression analysis is an established error [80] that had a substantial impact on the recently published report of the safety of acetaminophen use during pregnancy by Ahlqvist and colleagues [79]. Their initial analysis, corrected for sex and birth cohort only, showed an HR for high levels of acetaminophen use of 1.92 (CI = 1.65–2.22; *p* < 0.001). However, after correction for more than 20 inflammation-associated factors, the risk of heavy acetaminophen use was no longer significant (HR = 1.12; CI = 0.96–1.31; *p* = 0.17). Further, we show the considerable effects of underreporting of acetaminophen use in the Cox analysis, which causes a dramatic decrease in the calculated risk of using the drug compared to the actual risk, but not a complete loss of risk.

As pointed out in the Results, it is unknown whether or not prenatal use of acetaminophen was accurately reported in the Swedish dataset analyzed by Ahlqvist and colleagues [79]. We have previously observed wide ranges of reported pediatric acetaminophen use in various surveys involving the pediatric use of acetaminophen [2]. For example, Alemany and colleagues [87] found that, based on reports by mothers, 7.7% of children were exposed to acetaminophen prior to 18 months of age in the Danish National Birth cohort (DNBC). In contrast, a study of Danish children born during the same time period as those in the DNBC found that 65% of children were exposed to acetaminophen within a three-month period [88]. Given the importance of accurate documentation of acetaminophen use for accurate estimation of risks demonstrated in the analyses presented herein, questions about why some datasets contain unusually low reports of over-the-counter drug use merit investigation. Although speculative, it seems plausible that studies specifically asking individuals about the use of a diverse range of products containing acetaminophen (e.g., popular products in the US such as NyQuil^®^, Mucinex^®^, Goody’s^®^, Alka-Seltzer Plus^®^, etc.) will likely report much more use of the drug than studies asking about drug use in general without questions about use of specific products.

A high prevalence of acetaminophen use in a population can result in potentially misleading outcomes, even when the HR is measured accurately using the correct model. For example, the true hazard ratio for virtual ASD following acetaminophen use was 2.5 (HR = 2.5000 based on model design, calculated via Cox analysis at 2.461; CI = 2.265–2.675; *p* = 2 × 10^−16^) as a result of 60% virtual acetaminophen use causing 50% of all cases of virtual ASD. In contrast, the true hazard ratio for ASD as a result of 7.5% virtual acetaminophen use was 14.3 (HR = 14.3333 based on model design, calculated via Cox analysis at 14.48; CI = 13.52–15.50; *p* = 2 × 10^−16^) (Figure 4, left panel) under conditions in which virtual acetaminophen use induced the same number of virtual ASD cases as in our model with 60% acetaminophen use (HR = 2.5). That is to say, widespread use of acetaminophen in the population will reduce the measured HR, regardless of the percentage of cases of ASD induced by acetaminophen. While it is expected that a dramatically lower use of acetaminophen will result in a lower prevalence of ASD, this hypothetical comparison is useful in understanding the implications of relatively low HRs in the face of widespread acetaminophen use. While lower HRs as a result of a high prevalence of use of acetaminophen are both expected and accepted from a mathematical/statistical perspective, the relatively low magnitude of the calculated HR in the face of the widespread use of acetaminophen might result in underappreciation of the risks of acetaminophen use during neurodevelopment, as pointed out above at the beginning of the Discussion.

Almost all published analyses of cohort databases probing the impact of acetaminophen on neurodevelopment adjust for inflammation/oxidative stress-related factors. This flawed approach is common when assessing the role of acetaminophen in neurodevelopmental disorders. We argue here that multivariate analyses of information from large databases is of limited value for addressing questions regarding the impact of acetaminophen on neurodevelopment. First, the extensive use of acetaminophen by individuals not at risk for acetaminophen-induced neurodevelopmental injury can cause deceptively low risk estimates and, as discussed above, can even obscure risks completely under some conditions [1]. Second, cofactors that create susceptibility to acetaminophen-induced injury cannot be easily factored into the analysis, but raw, uncompensated hazard ratios are of limited utility for drawing conclusions. Such uncompensated hazard ratios only confirm what is already known: for example, that children with ASD tend to be sicker than neurotypical children with numerous co-morbid conditions. A third factor that limits the utility of a multivariate analysis for assessing risks of acetaminophen for neurodevelopment is that the immediate postpartum period is the time frame in which individuals are most sensitive to acetaminophen-induced injury. Conclusions regarding this window of sensitivity are based on pharmacokinetic considerations and other lines of evidence reviewed previously by us [1,2,3] and briefly in the Introduction. In addition, acetaminophen use during pregnancy may be associated with acetaminophen use during the peripartum period and in early childhood, potentially confounding any efforts to parse out exactly what risk exists at any given time using a multivariate analysis of information from large datasets. Further, factors affecting the susceptibility of individuals to acetaminophen-induced injury during pregnancy are likely to be correlated with postpartum risk factors. With this in mind, even if data were collected perfectly during the pregnancy period, and even if the hazards ratio for acetaminophen use was large based on a multivariate analysis, that hazard could be a reflection of acetaminophen exposure after birth. Thus, using a multivariate analysis of cohort data, it is not possible to determine whether or not acetaminophen induces neurological injury at the present time. That being said, if the raw (unadjusted for any confounding factor) HRs were near zero, the role of acetaminophen in the induction of neurodevelopmental injury would seem unlikely. This has not been the case in previously published work assessing data from human cohorts [79,89,90].

The myriad factors associated with an increased risk of ASD provide excellent insight into factors leading to the toxicity of acetaminophen within the context of neurodevelopment. For example, numerous factors associated with ASD can cause oxidative stress [77], a factor known to impede the metabolism of acetaminophen and result in the production of toxic metabolites of acetaminophen, primarily N-acetyl-*p*-benzoquinone imine (NAPQI). These observations, taken together, indicate that toxic metabolites of acetaminophen likely play a key role in neurodevelopmental injury, resulting in ASD. In another example, the genes associated with autophagy are known to play a role in ASD [91]. At the same time, laboratory animal studies show that acetaminophen induces apoptosis of cortical neurons [24]. Taken together, these observations suggest that problems with tissue remodeling following acetaminophen-induced neuronal cell death might play a role in the pathogenesis of some cases of ASD.

While the multifactorial components rendering acetaminophen dangerous for the developing brain provide insight into the pathogenesis of ASD, it is the prominent role of acetaminophen itself in the pathogenesis that is of immense practical and clinical significance. Indeed, an actionable approach for reducing the prevalence of ASD can be implemented immediately based on changes in the use of acetaminophen [3]. When considering changes to medical practice, exposure of neonates to acetaminophen is of particular concern. Pregnant women metabolize acetaminophen more efficiently than other adults [92], conferring a level of protection to the fetus. In contrast, after birth, it takes approximately 10 days before a major pathway of acetaminophen metabolism free of toxic metabolites, glucuronidation, becomes active [93]. Importantly, the dramatically increased risk of ASD associated with cord blood acetaminophen and with circumcision strongly support the neonatal period as the period of greatest sensitivity to acetaminophen-induced neurodevelopmental injury [7,8]. Fortunately, no long-term benefits of acetaminophen have been demonstrated in neonates [3], suggesting that eliminating neonatal exposure to acetaminophen can be considered without proven long-term benefits. In addition, when evaluating possible changes to medical practice, administration of acetaminophen to the mother in the hours before birth should be considered, since the birth process will leave the neonate alone to process any unmetabolized drug residues. Further changes in medical practice affecting children under 6 years of age should also be considered, and are expected to result in profoundly improved neurological outcomes [3]. Finally, of primary ethical importance is the imperative to notify caregivers of (a) the dangers of acetaminophen for neurodevelopment and (b) the generally protective nature of fevers as a component of the immune response [94,95,96].

The myriad of factors associated with the induction of ASD has led some to conclude that a single factor cannot be responsible for the profound rise in the prevalence of ASD. This non sequitur is refuted by overwhelming evidence demonstrating the role of acetaminophen in the pathogenesis of ASD (see the Introduction and our previous reviews [1,2,3]). Although the multifactorial model alone accounts for only a subset of observations, it is still important as part of the etiology of ASD (Figure 2), providing insight into the pathogenesis of ASD. At the same time, the prominent role of acetaminophen in the model explains known observations to a far greater degree and provides a clear path forward to alleviating the burden of ASD on future generations.

## Figures and Tables

**Table 1:** Results of Cox regression analysis with 60% actual acetaminophen (APAP) use, all documented, and 50% of all ASD induced by APAP use in the 1.39% of individuals with the highest risk. The number of cofactors included in the analysis is shown in column 1. A total of 10 cofactors were used to create the dataset.

Number Cofactors Included	Variable	HR (*p*-Value)	Lower-Upper 95% CI
0	APAP	2.461 (2 × 10^−16^)	2.675–2.265
3	Cofactor 1	1.219 (2 × 10^−16^)	1.241–1.198
3	Cofactor 2	1.197 (2 × 10^−16^)	1.217–1.176
3	Cofactor 3	1.186 (2 × 10^−16^)	1.207–1.166
3	APAP	1.844 (2 × 10^−16^)	2.007–1.694
9	Cofactor 1	1.250 (2 × 10^−16^)	1.275–1.231
9	Cofactor 2	1.210 (2 × 10^−16^)	1.233–1.192
9	Cofactor 3	1.203 (2 × 10^−16^)	1.227–1.185
9	Cofactor 4	1.221 (2 × 10^−16^)	1.245–1.203
9	Cofactor 5	1.215 (2 × 10^−16^)	1.238–1.197
9	Cofactor 6	1.235 (2 × 10^−16^)	1.260–1.217
9	Cofactor 7	1.216 (2 × 10^−16^)	1.240–1.199
9	Cofactor 8	1.227 (2 × 10^−16^)	1.251–1.208
9	Cofactor 9	1.215 (2 × 10^−16^)	1.238–1.197
9	APAP	0.905 (0.0264)	0.828–0.988

**Table 2:** Results of Cox regression analysis with 60% actual acetaminophen (APAP) use, 7.5% documented APAP use, and 50% of all ASD induced by APAP use in the 1.39% of individuals with the highest risk. The number of cofactors included in the analysis is shown in column 1. A total of 10 cofactors were used to create the dataset.

Number Cofactors Included	Variable	HR (*p*-Value)	Lower-Upper 95% CI
0	APAP	1.364 (8.13 × 10^−8^)	1.218–1.528
3	Cofactor 1	1.238 (2 × 10^−16^)	1.217–1.260
3	Cofactor 2	1.216 (2 × 10^−16^)	1.196–1.237
3	Cofactor 3	1.205 (2 × 10^−16^)	1.185–1.226
3	APAP	1.197 (0.00196)	1.068–1.341
9	Cofactor 1	1.250 (2 × 10^−16^)	1.229–1.272
9	Cofactor 2	1.210 (2 × 10^−16^)	1.190–1.230
9	Cofactor 3	1.203 (2 × 10^−16^)	1.183–1.224
9	Cofactor 4	1.221 (2 × 10^−16^)	1.200–1.242
9	Cofactor 5	1.215 (2 × 10^−16^)	1.194–1.236
9	Cofactor 6	1.235 (2 × 10^−16^)	1.214–1.256
9	Cofactor 7	1.216 (2 × 10^−16^)	1.196–1.237
9	Cofactor 8	1.227 (2 × 10^−16^)	1.206–1.248
9	Cofactor 9	1.215 (2 × 10^−16^)	1.194–1.235
9	APAP	0.992 (0.886)	0.885–1.111

**Table 3:** Limitations of multivariate analyses of cohort data in the determination of the contribution of acetaminophen to the etiology of ASD.

Distinct Problems Identifying the Contribution of Acetaminophen Exposure to the Prevalence of ASD Using a Multivariate Analysis	Result/Solution
Evaluation of databases that capture a limited time during neurodevelopment will capture only a fraction of the risk of acetaminophen exposure *. Measures of exposure during all relevant time periods (e.g., during pregnancy, immediately postpartum, between 12 and 18 months of age) are not available for any cohort.	Odds ratios calculated based on one time period underestimate the impact of acetaminophen on neurodevelopment; the effects of acetaminophen within one developmental period should be considered in conjunction with effects during other developmental periods for policy making purposes.
Heavy use of acetaminophen among non-sensitive individuals reduces the calculated risk of exposure.	Relatively low odds ratios (e.g., 1.2–2.0) merit policy change if exposure is prevalent.
Underreporting of acetaminophen use reduces the calculated risk of exposure.	Exposure determination should be rigorous.
Variables associated with sensitivity cannot be treated as confounding factors.	Model assumptions determine outcomes: Calculate risks using interacting variables.
Exposure during one time period (e.g., pregnancy) may correlate with exposure during other time periods (e.g., first 18 months of life).	Assessment of risk during one time period correlates to an unknown degree with risk at other time periods; risks during specific time periods cannot be reliably determined.
Some variables associated with sensitivity during one time period (e.g., pregnancy) correlate with or may be identical to variables associated with sensitivity during other time periods (e.g., first 18 months of life).	Assessment of exposure during one time period may correlate with exposure at other time periods; risks during specific time periods cannot be reliably determined.

* Sensitivity to acetaminophen-mediated neurodevelopmental injury occurs over a broad time frame, possibly starting during gestation and ending at approximately 5 years of age, with peak sensitivity occurring immediately postpartum [3]. The incidence of ASD is expected to be a sum of the products of exposures during various time periods and the factors dictating sensitivity during those time periods. That is, incidence = E_1_S_1_ + E_2_S_2_ + E_3_S_3_…, where E_x_ is the exposure of the population to acetaminophen during time period x and S_x_ is the relative sensitivity of the population to acetaminophen-mediated neurodevelopmental injury during time period x.

## Data Availability

The original contributions presented in the study are included in the article, further inquiries can be directed to the corresponding author.
